# Global burden and trends of hypertension-related chronic kidney disease attributable to high body mass index or low physical activity: an analysis based on global burden of disease study 2021 data

**DOI:** 10.3389/fnut.2025.1701077

**Published:** 2026-01-12

**Authors:** Xiaohua Lin, Kaiyi Miao, Kaiqi Huang, Yanfang Xu, Yujia Wang

**Affiliations:** 1 The First Clinical Medical College of Fujian Medical University, The First Affiliated Hospital of Fujian Medical University, Fuzhou, China; 2 Department of Nephrology, Blood Purification Research Center, The First Affiliated Hospital, Fujian Medical University, Fuzhou, China; 3 Research Center for Metabolic Chronic Kidney Disease, The First Affiliated Hospital, Fujian Medical University, Fuzhou, China; 4 Department of Nephrology, National Regional Medical Center, Binhai Campus of the First Affiliated Hospital, Fujian Medical University, Fuzhou, China

**Keywords:** hypertension-related chronic kidney disease, global burden of disease, body mass index, physical activity, disability-adjusted life years, metabolic risk factors

## Abstract

**Background:**

Hypertension-related chronic kidney disease (HT-CKD) represents a global health threat exacerbated by modifiable metabolic risks. Elevated body mass index (BMI) and low physical activity contribute to the development of hypertension and subsequent HT-CKD. This study quantified the global burden of HT-CKD attributable to elevated BMI or low physical activity from 1990 to 2021 and projected future trends to 2050.

**Methods:**

Based on the Global Burden of Disease (GBD) 2021 data, deaths, disability-adjusted life years (DALYs), and age-standardized rates (ASRs) of HT-CKD attributable to elevated BMI or physical inactivity were analyzed. Temporal trends of the disease burden from 1990 to 2021 were assessed using a linear regression model. Future burden was forecasted to 2050 using the autoregressive integrated moving average (ARIMA) model and the exponential smoothing (ES) model.

**Results:**

In 2021, HT-CKD attributable to elevated BMI caused 179,788 deaths and 4.26 million DALYs globally, representing increases of 392.9 and 322.2% since 1990. HT-CKD attributable to low physical activity caused 4,479 deaths and 77,879 DALYs, with similar substantial growth. ASRs for deaths and DALYs showed significant global upward trends from 1990 to 2021 for both risk factors. North Africa had the highest ASRs of death and DALYs for HT-CKD attributable to elevated BMI. For HT-CKD attributable to low physical activity, North Africa and South Africa recorded the highest ASRs of death and DALYs, respectively. The overall disease burden increased with age, peaking in populations aged ≥80 and reaching maximal ASRs in the ≥95-year age group for both metrics and risk factors. Gender disparities revealed higher absolute deaths and DALYs in men for HT-CKD attributable to elevated BMI, but ASR growth was rapid in women for HT-CKD attributable to low physical activity. Across all sociodemographic index (SDI) quintiles, disease burden increased significantly. High-SDI regions showed the steepest ASR growth for HT-CKD attributable to elevated BMI and to low physical activity, whereas low-SDI regions had the slowest growth. Both ARIMA and ES models indicated continued increases in deaths and DALYs attributable to both risk factors from 2022–2050, especially for elevated BMI.

**Conclusion:**

Elevated BMI is the dominant metabolic driver of HT-CKD burden, with physical inactivity playing a significant role. This burden demonstrates pronounced demographic and geographic disparities, highlighting the need for urgent, targeted public health interventions to combat obesity and promote physical activity.

## 1 Introduction

Chronic Kidney Disease (CKD) is a growing and formidable global public health challenge. Among its diverse etiologies, hypertension plays a pivotal role. Evidence demonstrates that blood pressure levels from high-normal to overt hypertension, constitute an independent risk factor for incident CKD, imposing a considerable disease burden on both male and female populations (1). Recent genomic studies in humans and animal models indicate that heritable CKD risk in hypertension stems from variations impacting glomerular/tubular protein handling, renal vascular autoregulation, and specifically, innate and adaptive immune mechanisms (2). Hypertension and CKD are mutually reinforcing: uncontrolled blood pressure accelerates intraglomerular hypertension, glomerulosclerosis, and tubulointerstitial injury (3), thereby propelling progression to end-stage renal disease (ESRD), while declining renal function amplifies blood pressure. This bidirectional, self-perpetuating loop establishes hypertension as the leading modifiable driver of adverse renal and cardiovascular outcomes in patients with CKD.

The surge of hypertension parallels a deteriorating metabolic milieu. Elevated body mass index (BMI) and low physical activity—two pivotal metabolic determinants—exacerbate hypertension and hypertension-related renal injury by potentiating renin-angiotensin-aldosterone system (RAAS) activation, sodium retention, and chronic low-grade inflammation, thereby increasing the global burden of hypertension-related chronic kidney disease (HT-CKD).

Obesity synergistically drives hypertension and renal injury through convergent pathways: visceral fat accumulation triggers sympathetic overdrive and up-regulates the RAAS, raising systemic vascular resistance and promoting sodium retention (4). Concomitantly, adipokine imbalance—hyperleptinaemia amplifies renal sympathetic excitation and inflammation, while declining adiponectin erodes endothelial protection—exacerbates vascular dysfunction and insulin resistance. Within the kidney, renal-sinus fat compresses tubular vasculature, activates tubuloglomerular feedback, and induces glomerular hyperfiltration and intrarenal hypertension. Lipotoxicity further stimulates activation of NLRP3, sustaining chronic low-grade inflammation and oxidative stress that accelerate podocyte injury and renal fibrosis (5).

Beyond obesity, physical inactivity independently increases the risk of hypertension and CKD. Low cardiorespiratory fitness predicts incident hypertension in prospective cohorts (6), while sedentary behavior heightens cardiovascular mortality and CKD progression (7). Inactivity blunts shear-stress–mediated nitric oxide bioavailability, elevates sympathetic tone, and impairs natriuresis, thereby fostering sodium retention and intraglomerular hypertension. Randomized trials confirm that substituting sedentary time with moderate-to-vigorous activity lowers systolic/diastolic BP by 11/5 mm Hg in hypertensive individuals and mitigates renal hyperfiltration (8).

The population-level contribution of elevated BMI and low physical activity to the global burden of HT-CKD remains poorly defined. Using GBD 1990–2021, we estimated the disease burden for HT-CKD attributable to these two modifiable metabolic risks and assessed the global temporal trends over three decades.

## 2 Methods

### 2.1 Data source and definitions

This study utilized data from GBD Study 2021. Data on the global burden of HT-CKD attributable to high BMI and low physical activity were sourced from the GBD 1990–2021. This comprehensive epidemiological repository that provides annual death counts, DALYs, and ASRs, systematically stratified based on demographic and geographic categories.

The two main metrics of HT-CKD attributable to elevated BMI and low physical activity focused in this study, include mortality and DALYs. Data across 1990–2021 were collected from the Global Health Data Exchange (GHDx), including annual statistics stratified by 12 five-year age bands (40–44, 45–49, 50–54, 55–59, 60–64, 65–69, 70–74, 75–79, 80–84, 85–90, 90–94, and ≥95 years), gender, 21 GBD regions, and five sociodemographic index (SDI) quintiles. This age threshold of≥40 years was selected because the HT-CKD burden is predominantly concentrated in this population, with comparatively fewer cases and lower attributable burden in younger age groups.

The GBD 2021 database identifies HT-CKD cases mainly based on the International Classification of Diseases, 10th Revision (ICD-10) (9, 10). Elevated BMI was defined as BMI ≥ 25 kg/m^2^ for adults aged ≥20 years, while the physical activity exposure was evaluated using metabolic equivalents (MET), defined as the ratio of caloric consumption during activity to basal metabolic rate at rest. LPA was defined as <3,000 MET min per week.

### 2.2 Estimation methods

Within the GBD comparative risk assessment framework, the burden attributable to each risk factor is estimated independently. This framework calculates the population attributable fraction (PAF) for each risk based on its theoretical minimum risk exposure level (TMREL), without accounting for potential interactions or overlaps between the two risk factors.

### 2.3 Data analysis

Descriptive burden: This study presented the numbers and rates of deaths and DALYs, each reported with 95% uncertainty intervals (UI). All burden evaluations were stratified by region, gender, and age group, and reported as counts and ASRs per 100,000 population for the years 1990 and 2021. The calculation of burden metrics was conducted using the standard GBD methodology (11). Mortality rates were calculated as the number of deaths in a specific age-gender-location-year group divided by the corresponding population estimate. DALYs were computed by summing years of life lost (YLLs) and years lived with disability (YLDs). The ASRs were calculated to facilitate comparisons across populations with differing age structures using the direct standardization method as given by $\mathrm{ASR}=\frac{\sum_{i=1}^{A}a_{i}w_{i}}{\sum_{i=1}^{A}w_{i}}\times 100,000$ , where *ai* denotes the age-specific rate for the *i*th age group, and *wi* refers to its corresponding weight in the reference standard population.

Temporal trends: Using ASRs from 1990–2021 ASRs, the log-linear regression models were assessed with natural logarithm of each rate. The estimated annual percentage change (EAPC) was calculated as [exp(β)- 1] × 100%, where β denotes the regression slope. Trends were considered statistically significant when the 95% confidence interval excluded zero.

Forecasting: Mortality and DALYs were projected to 2050 with ARIMA and ES models. The ARIMA model is particularly suited to capturing complex temporal structures, including autocorrelation, trend, and seasonality, by integrating differencing with autoregressive and moving average components (12). ES models assign exponentially decreasing weights to past observations, placing greater emphasis on recent data to enhance short- to medium-term forecast accuracy (13). The combined application of ARIMA and ES provides a robust framework for forecasting, as ARIMA excels in capturing long-term dependencies and seasonal patterns, whereas ES is highly adaptive to recent fluctuations and shifts in trends.

## 3 Results

### 3.1 Global burden of HT-CKD attributable to elevated BMI

#### 3.1.1 Disease burden in 2021

In 2021, HT-CKD attributable to elevated BMI resulted in 179,788 [95% uncertainty interval (UI): 91,323–260,251] deaths, accounting for 0.26% of global deaths. The corresponding age-standardized death rate was 2.19 (95% UI: 1.11–3.19) per 100,000 population. The number of DALYs was 4,257,037 (95% UI: 2,119,854–6,136,203), representing 6.17% of global DALYs. The age-standardized DALY rate was 50.11 (95% UI: 25.00–72.49) per 100,000 population (Tables 1, 2).

**Table 1:** The number of deaths cases and the age-standardized rate of hypertension-related CKD attributable to elevated BMI in 1990 and 2021, and its trends from 1990 to 2021 globally.

Characteristics	1990		2021		1990–2021
	Number of deaths cases(95% UI)	The age-standardized deaths rate/100000 (95% UI)	Number of deaths cases(95% UI)	The age-standardized deaths rate/100000 (95% UI)	EAPC(95% CI)
Global	36,476 (17,465, 59,219)	1.09 (0.52,1.74)	179,788 (91,323, 260,251)	2.19 (1.11, 3.19)	3.38 (2.99, 3.78)
Gender					
Male	17,805 (8,034, 30,386)	1.28 (0.60,2.11)	88,941 (44,216, 131,308)	2.53 (1.27, 3.73)	2.51 (2.38, 2.63)
Female	18,671 (9,115, 28,969)	0.97 (0.48, 1.49)	90,847 (47,294, 131,912)	1.95 (1.02, 2.82)	2.52 (2.36, 2.68)
Age					
40–44	938 (335, 1739)	0.33 (0.12, 0.61)	3,471 (1,415, 5,942)	0.69 (0.28, 1.19)	2.39 (2.29, 2.5)
45–49	1,262 (472, 2,391)	0.54 (0.20, 1.03)	5,039 (2,162, 8,888)	1.06 (0.46, 1.88)	2.21 (2.14, 2.28)
50–54	2001 (819, 3,659)	0.94 (0.39, 1.72)	8,119 (3,816, 13,585)	1.82 (0.86, 3.05)	2.18 (2.06, 2.3)
55–59	2,761 (1,156, 5,067)	1.49 (0.62, 2.74)	11,835 (5,622, 19,221)	2.99 (1.42, 4.86)	2.39 (2.24, 2.54)
60–64	3,455 (1,546, 6,088)	2.15 (0.96, 3.79)	14,578 (7,581, 23,286)	4.56 (2.37, 7.28)	2.47 (2.34, 2.6)
65–69	3,899 (1722, 7,084)	3.15 (1.39, 5.73)	17,469 (8,554, 27,253)	6.33 (3.10, 9.88)	2.43 (2.29, 2.56)
70–74	4,321 (1822, 7,673)	5.10 (2.15, 9.06)	20,366 (9,326, 32,080)	9.89 (4.53, 15.58)	2.38 (2.28, 2.49)
75–79	4,976 (2,215, 8,909)	8.08 (3.60, 14.47)	21,437 (10,200, 34,120)	16.25 (7.73, 25.87)	2.35 (2.27, 2.43)
80–84	4,693 (2,151, 8,006)	13.27 (6.08, 22.63)	22,559 (10,590, 36,129)	25.76 (12.09, 41.25)	2.44 (2.26, 2.62)
85–89	4,074 (1852, 7,025)	26.96 (12.25, 46.49)	24,047 (11,713, 38,553)	52.59 (25.62, 84.32)	2.71 (2.49, 2.93)
90–94	2014 (891, 3,445)	46.99 (20.78, 80.39)	17,571 (8,259, 28,587)	98.22 (46.17, 159.80)	2.99 (2.76, 3.21)
≥95	723 (313, 1,186)	71.04 (30.77, 116.48)	8,866 (3,725, 14,189)	162.67 (68.35, 260.34)	3.18 (3.02, 3.34)
SDI					
Low SDI	2,581 (1,175, 4,492)	1.37 (0.61, 2.40)	9,008 (4,324, 15,139)	2.10 (1.00, 3.49)	1.33 (1.20, 1.45)
Low-middle SDI	6,121 (2,851, 10,374)	1.20 (0.55, 2.02)	31,606 (16,127, 46,554)	2.46 (1.25, 3.64)	2.45 (2.36, 2.54)
Middle SDI	10,525 (4,690, 17,535)	1.29 (0.59, 2.15)	57,612 (27,792, 87,626)	2.38 (1.18, 3.65)	2.11 (1.90, 2.32)
High-middle SDI	7,334 (3,610, 11,665)	0.90 (0.45, 1.41)	28,394 (14,298, 42,277)	1.50 (0.75, 2.23)	1.87 (1.80, 1.95)
High SDI	9,869 (4,970, 15,206)	0.91 (0.46, 1.40)	52,995 (27,301, 74,271)	2.19 (1.16, 2.99)	3.43 (3.22, 3.65)

UI, uncertainty intervals; CI, confidence interval; SDI, sociodemographic index; EAPC, estimated annual percentage change.

**Table 2:** The number of disability-adjusted-life-years (DALYs) cases and the age-standardized rate of hypertension-related chronic kidney diseases (HT-CKD) attributable to elevated body mass index (BMI) in 1990 and 2021, and its trends from 1990 to 2021 globally.

Characteristics	1990		2021		1990–2021
	Number of DALYs cases(95% UI)	The age-standardized DALYs rate/100000 (95% UI)	Number of DALYs cases(95% UI)	The age-standardized DALYs rate/100000 (95% UI)	EAPC(95% CI)
**Global**	1,008,372 (468,964, 1,610,433)	26.04 (12.18, 41.16)	4,257,037 (2,119,854, 6,136,203)	50.11 (25.00, 72.49)	2.97 (2.7,3.23)
Gender					
Male	514,524 (235,951, 864,141)	29.38 (13.51, 48.60)	2,213,202 (1,082,658, 3,225,998)	56.64 (27.90, 82.48)	2.34 (2.24,2.45)
Female	493,848 (234,469, 757,913)	23.64 (11.24, 36.32)	2,043,835 (1,032,283, 2,932,756)	44.76 (22.54, 64.07)	2.22 (2.09,2.35)
Age					
40–44	57,969 (22,859, 101,012)	20.23 (7.98, 35.26)	203,905 (88,115, 331,238)	40.76 (17.61, 66.21)	2.27 (2.17,2.36)
45–49	67,844 (27,538, 119,716)	29.22 (11.86, 51.56)	260,827 (116,356, 433,648)	55.08 (24.57, 91.58)	2.11 (2.03,2.19)
50–54	92,202 (38,800, 165,068)	43.37 (18.25, 77.65)	362,243 (174,521, 596,872)	81.42 (39.22, 134.15)	2.1 (1.98,2.23)
55–59	109,735 (47,269, 194,049)	59.25 (25.25, 104.78)	454,926 (224,908, 717,335)	114.96 (56.83, 181.27)	2.3 (2.16,2.45)
60–64	118,427 (55,206, 202,564)	73.74 (34.37, 126.12)	478,896 (248,804, 744,033)	149.63 (77.74, 232.48)	2.37 (2.25,2.50)
65–69	112,933 (51,602, 197,391)	91.36 (41.75, 159.69)	484,090 (243,526, 747,985)	175.50 (88.28, 271.16)	2.31 (2.18,2.43)
70–74	101,651 (45,031, 174,985)	120.07 (53.19, 206.69)	462,146 (218,735, 713,465)	224.52 (106.26, 346.61)	2.26 (2.15,2.36)
75–79	94,475 (45,080, 163,350)	153.48 (73.23, 265.37)	388,524 (190,995, 612,832)	294.59 (144.82, 464.67)	2.21 (2.12,2.30)
80–84	70,452 (32,590, 117,017)	199.15 (92.12, 330.78)	320,906 (156,895, 505,574)	366.40 (179.14, 577.25)	2.24 (2.07,2.42)
85–89	48,368 (22,186, 83,237)	320.08 (150.79, 550.83)	267,510 (133,164, 428,263)	585.08 (291.25, 936.67)	2.44 (2.24,2.64)
90–94	21,148 (9,781, 34,968)	493.51 (228.26, 816.02)	170,097 (80,416, 271,103)	950.83 (449.52, 1515.44)	2.64 (2.45,2.84)
≥95	6,975 (3,071, 11,293)	685.08 (301.63, 1109.23)	78,456 (34,114, 122,693)	1439.48 (625.91, 2251.13)	2.84 (2.7,2.97)
SDI					
Low SDI	75,790 (33,397, 131,330)	32.40 (14.58, 55.39)	267,387 (131,391, 449,180)	49.19 (23.82, 82.87)	1.25 (1.15,1.35)
Low-middle SDI	180,272 (82,102, 299,057)	28.87 (13.44, 47.91)	881,403 (455,362, 1,285,125)	59.68 (30.18, 86.75)	2.44 (2.34,2.54)
Middle SDI	313,079 (138,517, 507,808)	30.23 (13.51, 49.56)	1,484,742 (719,476, 2,261,098)	55.79 (26.83, 84.40)	2.06 (1.85,2.28)
High-middle SDI	200,215 (97,807, 313,072)	21.43 (10.52, 33.65)	616,991 (309,422, 896,716)	32.26 (16.20, 47.00)	1.49 (1.44,155)
High SDI	237,694 (120,680, 353,159)	22.05 (11.13, 32.55)	1,002,356 (541,756, 130)	48.90 (26.93, 61.49)	3.05 (2.89,3.21)

DALYs, disability-adjusted-life-years; UI, uncertainty intervals; CI, confidence interval; SDI, sociodemographic index; EAPC, Estimated annual percentage change.

#### 3.1.2 Region and country subgroup analyses

**Figure 1:**
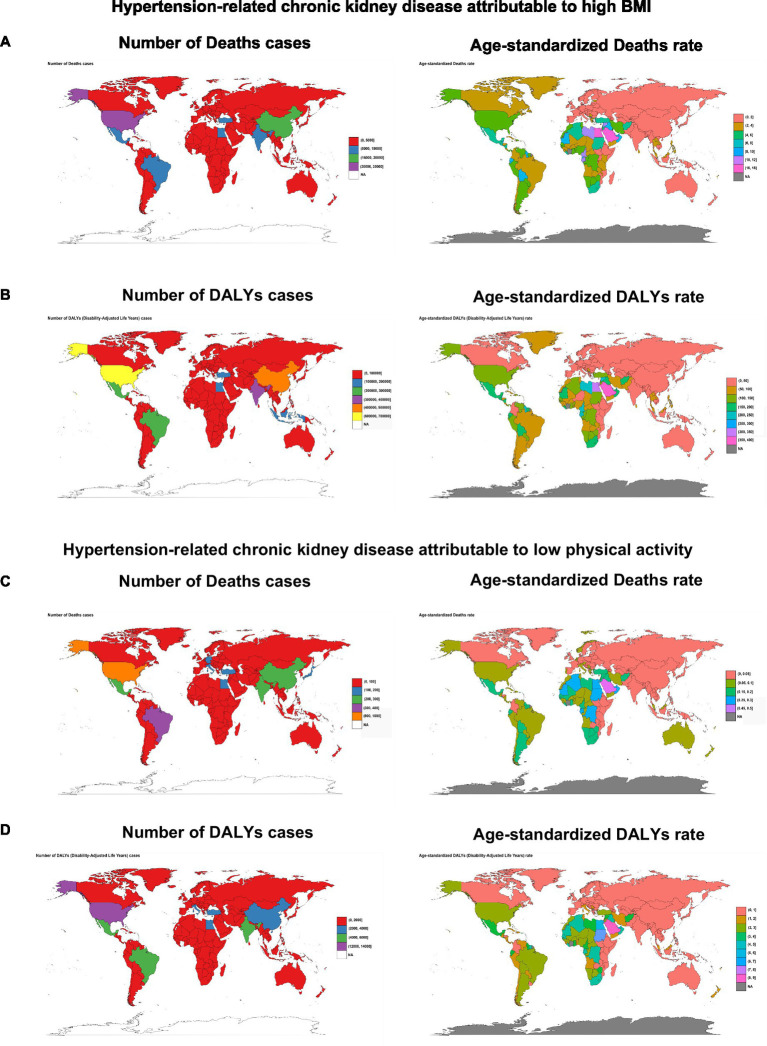
The global and regional distribution of death, disability-adjusted life years (DALYs), and corresponding age-standardized rates (ASRs) of hypertension-related chronic kidney disease (HT-CKD) attributable to elevated body mass index (BMI) or low physical activity in 2021. Numbers and ASRs of deaths **(A)** and DALYs **(B)** for HT-CKD attributable to high BMI, as well as numbers and ASRs of deaths **(C)** and DALYs **(D)** for HT-CKD attributable to low physical activity, across countries and territories in 2021. DALYs, disability-adjusted life years.

Globally, the burden of HT-CKD attributable to elevated BMI exhibited substantial geographical heterogeneity (Figure 1). Among all regions, the Americas recorded the highest absolute death count (66,801; 95% UI: 36,624–90,568), while Asia carried the highest DALYs burden (1,627,931; 95% UI: 724,859–2,646,015) in 2021. Oceania demonstrated the lowest absolute burden for both deaths (60; 95% UI: 27–109) and DALYs (2,221; 95% UI: 991–3,900). ASRs revealed divergent patterns: North Africa showed the highest death ASR (9.64/100,000; 95% UI: 5.06–13.70) and DALYs ASR (187.86/100,000; 95% UI: 103.78–258.44), whereas Central Asia had the lowest death ASR (0.31/100,000; 95% UI: 0.17–0.46) and high-income Asia Pacific had the lowest DALYs ASR (11.28/100,000; 95% UI: 5.40–18.34).

Country-level analysis mirrored this heterogeneity. The United States demonstrated the highest absolute burden with 32,009 deaths (95% UI: 16,399–44,232) and 600,505 DALYs (95% UI: 320,531–762,094), followed by China. Microstates exhibited minimal burdens, with Tokelau recording fewer than two cases for both deaths and DALYs (95% UI: 0–1). Saudi Arabia demonstrated the highest ASRs for deaths (17.75/100,000; 95% UI: 9.62–24.68) and DALYs (355.26/100,000; 95% UI: 193.62–490.42), followed by Egypt. In contrast, Tajikistan exhibited the lowest death ASR (0.06/100,000; 95% UI: 0.03–0.09), while Finland had the lowest DALYs ASR (8.83/100,000; 95% UI: 4.72–13.28).

#### 3.1.3 Temporal trends from 1990 to 2021

**Figure 2:**
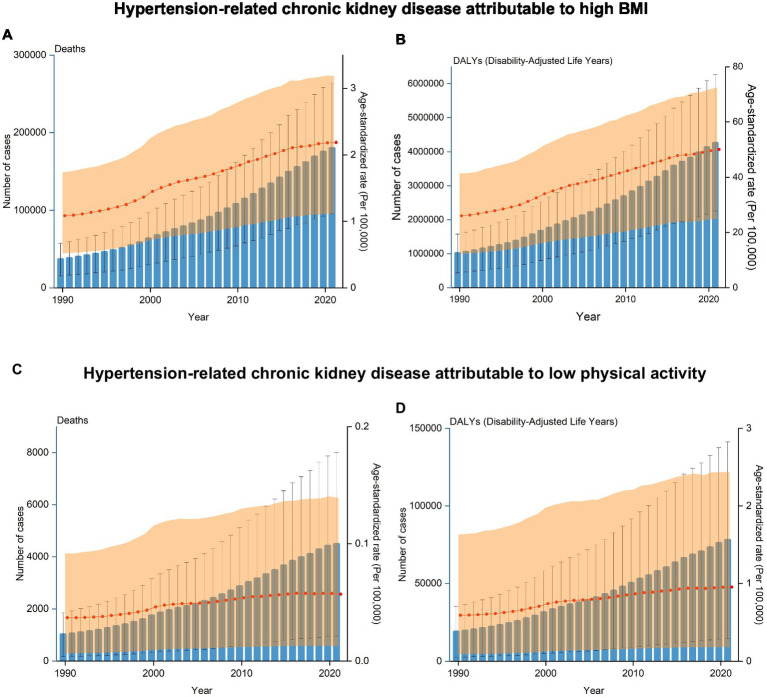
Trends in deaths and disability-adjusted life years (DALYs) rates of hypertension-related chronic kidney disease (HT-CKD) attributable to elevated body mass index (BMI) or low physical activity from 1990 to 2021. The trends in the numbers and ASRs of deaths **(A)** and DALYs **(B)** for HT-CKD attributable to high BMI, as well as the trends in the numbers and ASRs of deaths **(C)** and DALYs **(D)** for HT-CKD attributable to low physical activity from 1990 to 2021.

The number of deaths from HT-CKD attributable to elevated BMI increased from 36,476 cases in 1990 to 179,788 cases in 2021, representing a 392.89% increase. Similarly, the corresponding age-standardized death rate showed consistent growth, increasing by 100.92%. The number of DALY cases and the ASR also followed the same pattern. DALY cases increased from 1,008,372 in 1990 to 4,257,037 in 2021 (a 322.17% increase), whereas, in the case of ASR, it increased from 26.04 to 50.11, reflecting a 92.43% growth. During the study period, the global age-standardized death rates and DALYs showed a significant upward trend with the EAPCs of 3.38% (95% confidence interval (CI): 2.99–3.78%) for death rates and 2.97% (95% CI: 2.7–3.23%) for DALYs (Tables 1, 2). An analysis of annual trends in deaths and DALYs from 1990 to 2021 demonstrated a consistent year-on-year growth pattern (Figure 2).

#### 3.1.4 Gender subgroup analyses

**Figure 3:**
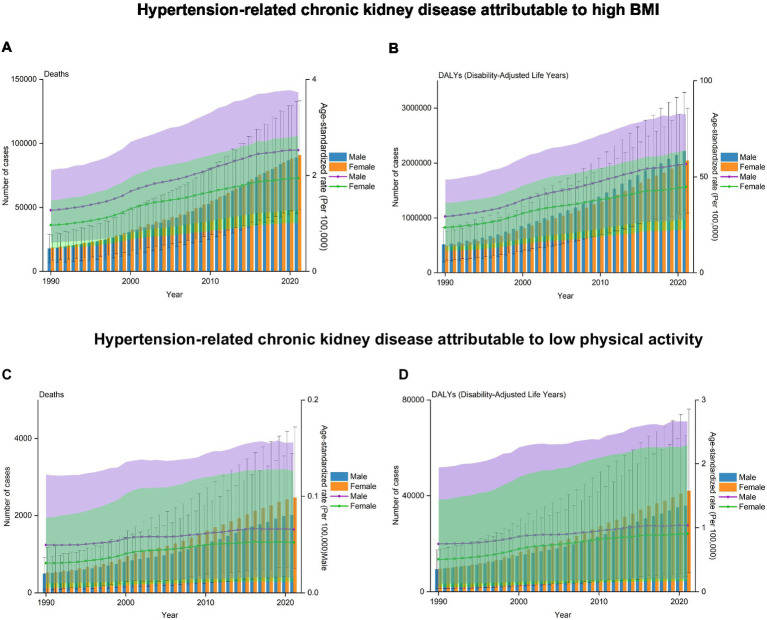
Trends in deaths and DALYs rates of HT-CKD attributable to elevated body mass index (BMI) or low physical activity by gender from 1990 to 2021. The trends in numbers and ASRs of deaths **(A)** and DALYs **(B)** for HT-CKD attributable to high BMI, as well as numbers and ASRs of deaths **(C)** and DALYs **(D)** for HT-CKD attributable to low physical activity, across gender (both male and female), from 1990 to 2021.

Global trends by gender over the study period indicated that the number of deaths and DALYs of HT-CKD attributable to elevated BMI increased annually in both male and female individuals. Specifically, global deaths due to HT-CKD attributable to elevated BMI increased from 17,805 (95% UI: 8,034–30,806) in 1990 to 88,941 (95% UI: 44,216–131,308) in 2021 among male individuals, and from 18,671 (95% UI: 9,115–28,969) in 1990 to 90,847 (95% UI: 47,294–131,912) in 2021 among female individuals. This represented a nearly 5.0-fold increase for men and a 4.9-fold increase for women (Figure 3; Table 1). The patterns of DALYs mirrored those of deaths, with a nearly 3.3-fold increase (from 514,524 to 2,213,202) for men and a 4.1-fold increase (from 493,848 to 2,043,835) for women during the same period (Figure 3; Table 2). The age-standardized death rates and DALYs demonstrated consistent upward trends over the study period, with EAPCs of 2.51% (95% CI: 2.38–2.63%) in males and 2.52% (95% CI: 2.32.68%) in females for death rates, and 2.34% (95% CI: 2.24–2.45%) in males and 2.22% (95% CI: 2.09–2.35%) in females for DALYs (Tables 1, 2). These findings underscore the sustained and significant annual increases in both mortality and disease burden attributable to HT-CKD due to elevated BMI across both genders.

#### 3.1.5 Age subgroup analyses

**Figure 4:**
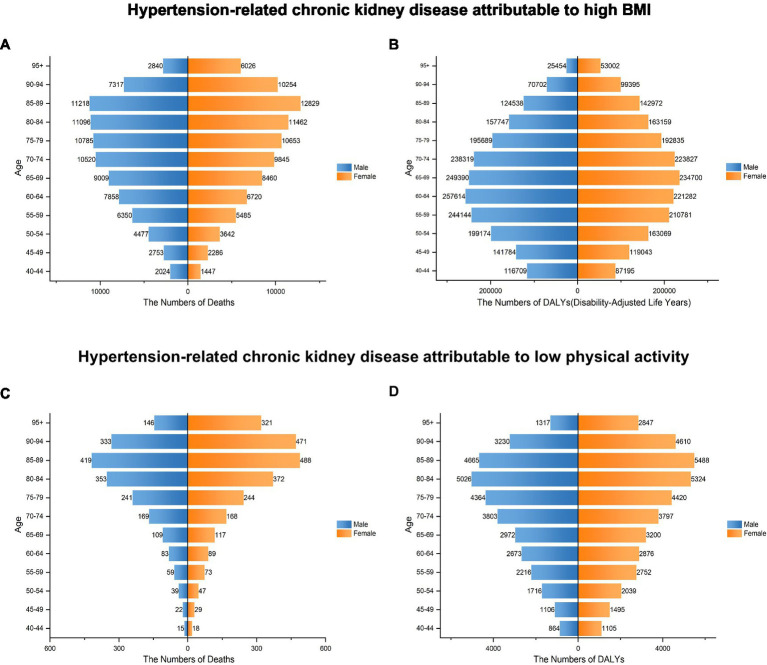
Distribution of deaths and DALYs rates of HT-CKD attributable to high BMI or low physical activity by age group and gender in 2021. Numbers of deaths **(A)** and DALYs **(B)** for HT-CKD attributable to high BMI, as well as numbers of deaths **(C)** and DALYs **(D)** for HT-CKD attributable to low physical activity, across gender (both male and female), by age groups ranging from 40 years to 95 + years, in 2021.

In 2021, the number of deaths and DALYs of HT-CKD attributable to elevated BMI increased progressively with age, beginning from 40 years and peaking in older age groups. This trend was consistent across both genders (Figure 4; Tables 1, 2). For mortality, peaks occurred in the 85–89 age group for both males and females. Male deaths exceeded female deaths till the 75–79 age group, after which females exhibited higher mortality in ages ≥80 years. Regarding DALYs, peaks occurred earlier: males peaked in the 60–64 age group and females in the 65–69 age group, with the male peak exceeding the female peak. DALYs demonstrated higher in males than females until the 75–79 age group, but were higher in females at ages ≥80 years. ASRs of deaths and DALYs demonstrated a stable upward trend from 1990 to 2021. The ≥95-year age group exhibited the highest rates, with deaths increasing from 71.04 (95% UI: 30.77–116.48) to 162.67 (95% UI: 68.35–260.34) and DALYs from 685.08 (95% UI: 301.63–1,109.23) to 1,439.48 (95% UI: 625.91–2,251.13), representing 2.3-fold and 2.1-fold increases, respectively. Further, this group exhibited highest growth rates in mortality (EAPC: 3.18; 95% UI: 3.02–3.34) and DALYs (EAPC: 2.84; 95% UI: 2.70–2.97). In contrast, the 50–54 age group exhibited the lowest growth rates for deaths (EAPC: 2.18; 95% UI: 2.06–2.30) and DALYs (EAPC: 2.10; 95% UI: 1.98–2.23) (Tables 1, 2). These analyses reveal age-specific disparities in elevated-BMI-attributable CKD burden.

#### 3.1.6 SDI subgroup analyses

From 1990 to 2021, all SDI groups demonstrated significant increases in both absolute numbers and ASRs of deaths and DALYs of HT-CKD attributable to elevated BMI. Middle SDI regions demonstrated the largest absolute growth in deaths, rising from 10,525 (95% UI: 4,690–17,535) to 57,612 (95% UI: 27,792–87,626), while low SDI regions showed the smallest increase [2,581 (95% UI: 1,175–4,492) to 9,008 (95% UI: 4,324–15,139)]. DALYs followed identical patterns, with middle SDI regions increasing most substantially [313,079 (95% UI: 138,517–507,808) to 1,484,742 (95% UI: 719,476–2,261,098)] and low SDI regions least [75,790 (95% UI: 33,397–131,330) to 267,387 (95% UI: 131,391–449,180)].

**Figure 5:**
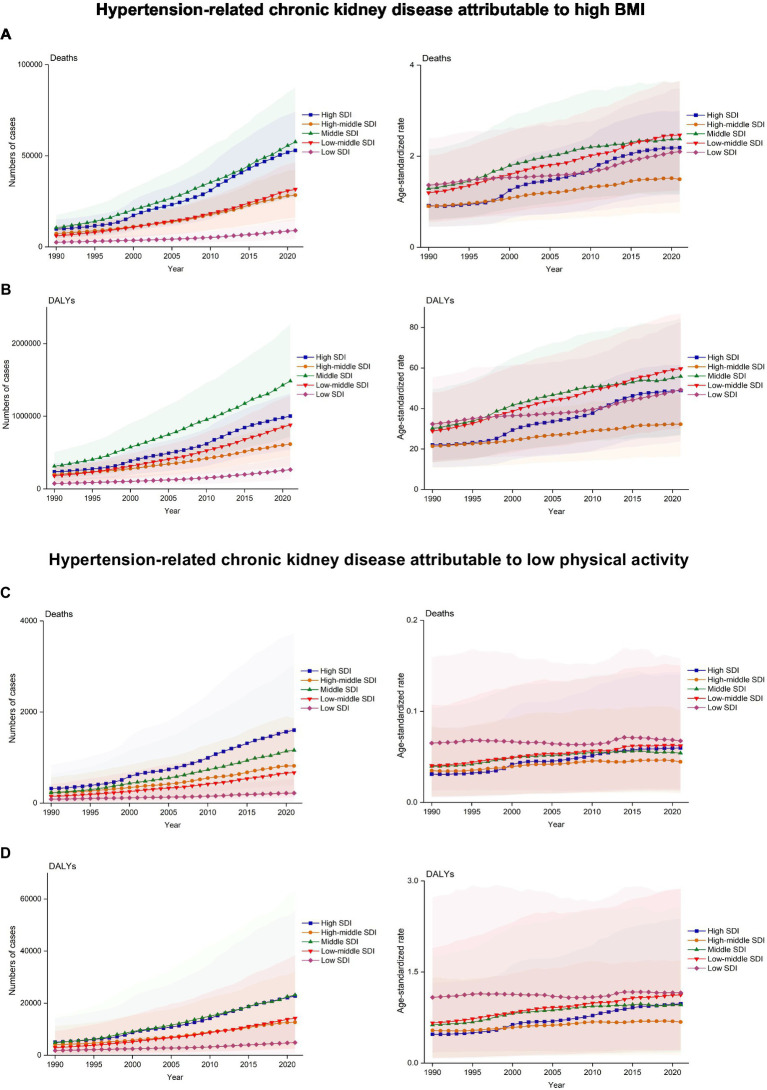
Trends in Deaths and DALYs rates of HT-CKD attributable to high BMI or low physical activity by sociodemographic index (SDI) from 1990 to 2021. The trends in numbers and ASRs of deaths **(A)** and DALYs **(B)** for HT-CKD attributable to high BMI, as well as numbers and ASRs of deaths **(C)** and DALYs **(D)** for HT-CKD attributable to low physical activity, across countries and territories by SDI, from 1990 to 2021.

Regarding ASR trends, the high SDI regions exhibited the steepest increase in mortality rate (0.91 to 2.19/100,000), while the low-middle SDI regions showed the greatest DALYs ASR growth (28.87 to 59.68/100,000). In contrast, middle-high SDI regions exhibited the smallest ASR changes for both deaths (1.37 to 2.10/100,000) and DALYs (21.43 to 32.26/100,000). Growth rate analysis demonstrated high SDI regions with the highest EAPCs [deaths: EAPC 3.43 (95% UI: 3.22–3.65); DALYs: 3.05 (2.89–3.21)], while low SDI regions had the lowest [deaths: EAPC 1.33 (1.20–1.45); DALYs: 1.25 (1.15–1.35)] (Figure 5; Tables 1, 2).

#### 3.1.7 Forecasting the future burden of HT-CKD attributable to elevated BMI

**Figure 6:**
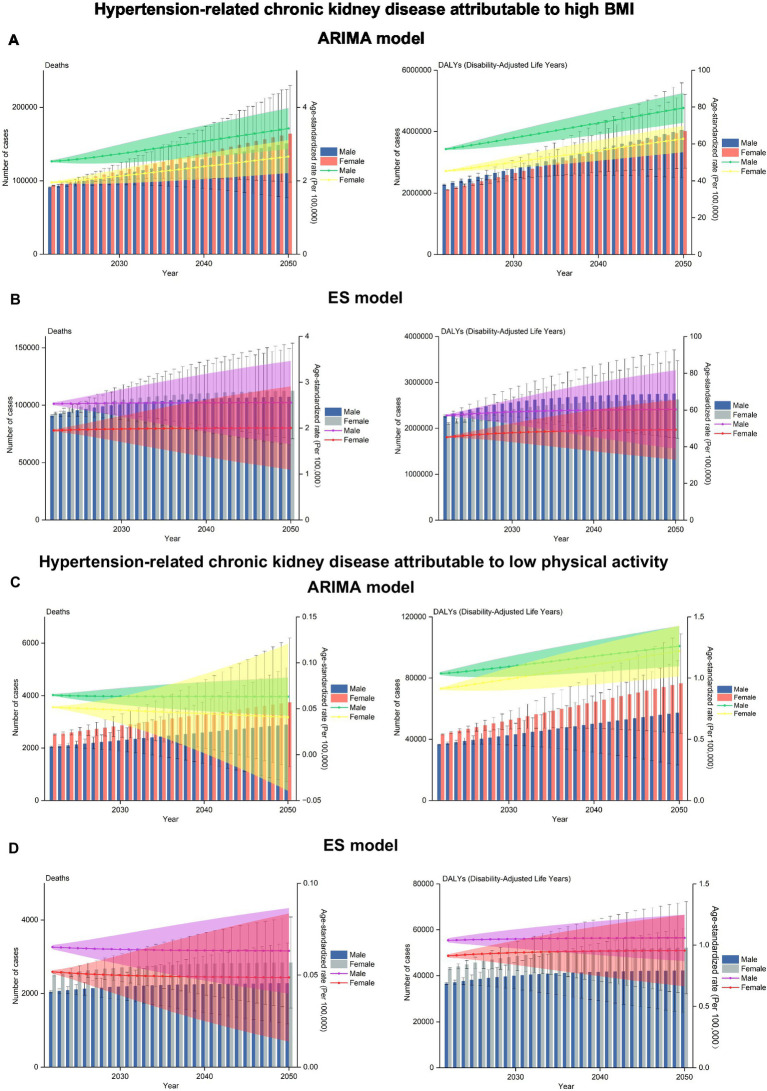
Projected deaths and DALYs for HT-CKD attributable to high BMI and low physical activity from 2022 to 2050, using the autoregressive integrated moving average (ARIMA) and the exponential smoothing (ES) models. The predicted results in numbers and ASRs of deaths and DALYs for HT-CKD attributable to high BMI are shown using the ARIMA model in **(A)** and the ES model in **(B)**. Correspondingly, projections for HT-CKD attributable to low physical activity are presented using the ARIMA model in **(C)** and the ES model in **(D)**.

The ARIMA and ES models were utilized to quantitatively describe the trend in deaths and DALYs from HT-CKD attributable to elevated BMI from 2022 to 2050 (Figure 6). The ARIMA predicted that from 2022 to 2050, deaths and DALYs may increase for both men (by 65.43 and 77.71%, respectively) and women (by 75.54 and 90.07%, respectively). The corresponding ASRs were projected to rise for both men (by 35.18 and 39.06%, respectively) and women (by 35.71 and 38.53%, respectively). From a gender perspective, increases in deaths and DALYs were lower among men than women, while corresponding ASRs were similar between genders. In contrast, the ES projections suggested that deaths and DALYs would rise for both men and women from 2020 through 2040 before stabilizing through 2050. Consistent ASRs for deaths and DALYs were expected to remain relatively stable during this period. Both models showed higher DALYs but lower deaths among men compared to women. Corresponding ASRs, however, were consistently higher for men than women.

### 3.2 Global burden of HT-CKD attributable to low physical activity

#### 3.2.1 Disease burden in 2021

In 2021, HT-CKD attributable to low physical activity exhibited in 4,479 (95% UI: 953–10,976) deaths, accounting for 0.0066% of global deaths. The corresponding age-standardized death rate was 0.057 (95% UI: 0.013–0.139) per 100,000 population. The number of DALYs was 77,879 (95% UI: 14,461–201,506), representing 0.1147% of global DALYs. The age-standardized DALY rate was 0.952 (95% UI: 0.180–2.437) per 100,000 population (Tables 3, 4).

**Table 3:** The number of deaths cases and the age-standardized rate of hypertension-related chronic kidney diseases (HT-CKD) attributable to low physical activity in 1990 and 2021, and its trends from 1990 to 2021 globally.

Characteristics	1990		2021		1990–2021
	Number of deaths cases(95% UI)	The age-standardized deaths rate/100000 (95% UI)	Number of deaths cases(95% UI)	The age-standardized deaths rate/100000 (95% UI)	EAPC(95% CI)
Global	1,013 (160, 2,606)	0.037 (0.007, 0.092)	4,479 (953, 10,976)	0.057 (0.013, 0.139)	2.76 (2.2,3.34)
Gender					
Male	492 (77, 1,281)	0.049 (0.010, 0.123)	2015 (421, 4,963)	0.065 (0.015, 0.156)	1.11 (1.03,1.20)
Female	521 (78, 1,382)	0.030 (0.005, 0.078)	2,464 (540, 5,974)	0.052 (0.011, 0.126)	2.03 (1.81,2.24)
Age					
40–44	6 (0, 24)	0.0023 (0.000065, 0.0083)	33 (2, 110)	0.0067 (0.00033, 0.0220)	3.56 (3.39,3.74)
45–49	9 (0, 32)	0.0038 (0.000097, 0.0137)	51 (3, 160)	0.0108 (0.00061, 0.0340)	3.45 (3.39,3.51)
50–54	16 (1, 62)	0.0074 (0.00031, 0.0292)	86 (7, 263)	0.0194 (0.0015, 0.0592)	3.21 (3.07,3.35)
55–59	26 (1, 99)	0.0138 (0.00061, 0.0532)	132 (8, 418)	0.0334 (0.0021, 0.110)	3.07 (2.83,3.3)
60–64	38 (1, 153)	0.0236 (0.00060, 0.0951)	172 (8, 538)	0.0539 (0.0025, 0.168)	2.68 (2.45,2.91)
65–69	54 (2, 204)	0.0439 (0.0013, 0.165)	226 (12, 702)	0.082 (0.0042, 0.255)	2.15 (1.95,2.35)
70–74	85 (4, 282)	0.101 (0.0042, 0.333)	337 (19, 998)	0.164 (0.009, 0.485)	1.68 (1.57,1.80)
75–79	149 (9, 483)	0.242 (0.015, 0.785)	485 (54, 1,367)	0.367 (0.041, 1.037)	1.31 (1.24,1.38)
80–84	209 (18, 595)	0.591 (0.050, 1.682)	725 (102, 1817)	0.828 (0.116, 2.075)	1.24 (1.08,1.39)
85–89	217 (29, 561)	1.439 (0.191, 3.715)	907 (186, 2,133)	1.985 (0.407, 4.664)	1.43 (1.24,1.63)
90–94	133 (24, 316)	3.097 (0.551, 7.373)	804 (203, 1808)	4.491 (1.113, 10.104)	1.68 (1.46,1.89)
≥95	56 (11, 129)	5.471 (1.092, 12.66)	467 (123, 1,073)	8.567 (2.260, 19.680)	1.86 (1.71,2.02)
SDI					
Low SDI	86 (13, 216)	0.07 (0.01, 0.16)	221 (41, 535)	0.07 (0.01, 0.16)	0.14 (0.01,0.26)
Low-middle SDI	148 (18, 412)	0.04 (0.01, 0.11)	670 (127, 1,670)	0.06 (0.01, 0.15)	1.51 (1.41,1.62)
Middle SDI	228 (33, 633)	0.04 (0.01, 0.10)	1,161 (220, 3,057)	0.05 (0.01, 0.14)	1.2 (1.01,1.38)
High-middle SDI	230 (39, 568)	0.04 (0.01, 0.08)	818 (183, 1856)	0.04 (0.01, 0.10)	1.09 (0.96,1.22)
High SDI	320 (51, 848)	0.03 (0.01, 0.08)	1,603 (395, 3,720)	0.06 (0.01, 0.14)	1.53 (1.33,1.73)

**Table 4:** The number of disability-adjusted life years (DALYs) cases and the age-standardized rate of hypertension-related chronic kidney diseases (HT-CKD) attributable to low physical activity in 1990 and 2021, and its trends from 1990 to 2021 globally.

Characteristics	1990		2021		1990–2021
	Number of DALYs cases(95% UI)	The age-standardized DALYs rate/100000 (95% UI)	Number of DALYs cases(95% UI)	The age-standardized DALYs rate/100000 (95% UI)	EAPC(95% CI)
Globa**l**	18,881 (2,527, 55,718)	0.593 (0.091, 1.631)	77,879 (14,461, 201,506)	0.952 (0.180, 2.437)	2.65 (2.22, 3.08)
Gender					
Male	9,364 (1,272, 27,078)	0.749 (0.128, 1.945)	35,873 (6,438, 95,864)	1.037 (0.199, 2.665)	1.21 (1.14, 1.29)
Female	9,517 (1,272, 28,246)	0.506 (0.074, 1.441)	42,006 (7,874, 105,694)	0.907 (0.169, 2.298)	2.1 (1.92, 2.27)
Age					
40–44	391 (12, 1,544)	0.136 (0.004, 0.539)	1970 (108, 6,300)	0.394 (0.022, 1.259)	3.55 (3.41, 3.69)
45–49	464 (12, 1,646)	0.200 (0.005, 0.709)	2,601 (140, 8,195)	0.549 (0.030, 1.731)	3.39 (3.33.3.45)
50–54	702 (30, 2,765)	0.330 (0.014, 1.301)	3,755 (284, 11,683)	0.844 (0.061, 2.626)	3.14 (2.99, 3.29)
55–59	991 (43, 3,810)	0.535 (0.023, 2.057)	4,968 (300, 15,921)	1.255 (0.076, 4.023)	3 (2.78, 3.22)
60–64	1,264 (32, 5,236)	0.787 (0.020, 3.260)	5,548 (254, 18,189)	1.734 (0.079, 5.683)	2.6 (2.38, 2.82)
65–69	1,533 (49, 5,633)	1.240 (0.040, 4.557)	6,172 (324, 19,163)	2.238 (0.098, 7.749)	2.06 (1.87, 2.25)
70–74	1966 (83, 6,560)	2.322 (0.098, 7.749)	7,600 (431, 22,574)	3.692 (0.209, 10.967)	1.62 (1.51, 1.73)
75–79	2,786 (172, 8,787)	4.527 (0.279, 14.275)	8,784 (965, 24,960)	6.660 (0.731, 18.925)	1.23 (1.15, 1.31)
80–84	3,109 (261, 8,816)	8.789 (0.737, 24.920)	10,350 (1,508, 26,557)	11.817 (1.722, 30.323)	1.09 (0.94, 1.25)
85–89	2,573 (351, 6,598)	17.028 (2.320, 43.662)	10,153 (2096, 23,711)	22.206 (4.585, 51.860)	1.2 (1.02, 1.37)
90–94	1,394 (246, 3,353)	32.521 (5.741, 78.254)	7,840 (1979, 17,509)	43.825 (11.062, 97.876)	1.37 (1.18, 1.56)
≥95	538 (111, 1,215)	52.863 (10.862, 119.335)	4,164 (1,099, 9,460)	76.395 (20.170, 173.574)	1.55 (1.42, 1.68)
SDI					
Low SDI	1852 (231, 5,037)	1.082 (0.174, 2.729)	4,906 (768, 13,012)	1.159 (0.216, 2.864)	0.14 (0.05, 0.23)
Low-middle SDI	2,989 (325, 9,406)	0.663 (0.083, 1.895)	14,225 (2,373, 38,440)	1.123 (0.209, 2.872)	1.78 (1.68, 1.88)
Middle SDI	4,861 (647, 14,491)	0.632 (0.093, 1.753)	23,149 (3,869, 63,144)	0.958 (0.170, 2.548)	1.49 (1.28, 1.69)
High-middle SDI	4,083 (581, 11,155)	0.538 (0.092, 1.407)	12,719 (2,514, 32,222)	0.677 (0.136, 1.692)	1.01 (0.91, 1.11)
High SDI	5,063 (684, 14,329)	0.477 (0.070, 1.322)	22,795 (4,708, 55,574)	0.979 (0.185, 2.383)	2.67 (2.53, 2.82)

#### 3.2.2 Region and country subgroup analyses

Globally, the burden of HT-CKD attributable to low physical activity exhibited substantial geographical heterogeneity (Figure 1). Among 50 regions, the Americas recorded the highest absolute death count (1,789; 95% UI: 419–3,978) and DALYs (29,450 95% UI: 5,991–69,832), while Asia followed closely behind. Oceania reported the lowest absolute burden for both deaths (1; 95% UI: 0–3) and DALYs (23; 95% UI: 1–83). ASRs revealed divergent patterns: North Africa showed the highest death ASR (0.236/100,000; 95% UI: 0.0547–0.510), and South Africa showed the highest DALYs ASR (4.209/100,000; 95% UI: 1.506–8.817). In contrast, Central Asia had the lowest death ASR (0.00386/100,000; 95% UI: 0.000454–0.0103), while Eastern Sub-Saharan Africa had the lowest DALYs ASR (0.157/100,000; 95% UI: 0.00722–0.597).

Country-level analysis showed significant global differences in the burden of HT-CKD attributable to low physical activity. In absolute terms, the United States reported the highest burden with 924 deaths (95% UI: 234–2,040) and 12,810 DALYs (95% UI: 2,730–30,718), followed by Brazil. Microstates exhibited minimal burdens, with Tokelau recording nearly zero cases for both deaths and DALYs. Regarding ASRs, Qatar had the highest death ASR at 0.515 per 100,000 (95% UI: 0.163–1.012), while Saudi Arabia had the highest DALYs ASR at 8.790 per 100,000 (95% UI: 1.931–19.583). In contrast, the Republic of Tajikistan reported the lowest death ASR of 0.000594 (95% UI: 0.0000645–0.00167), and Ethiopia had the lowest DALYs ASR at 0.0224 (95% UI: 0–0.209), with Eritrea also among the lowest for DALYs ASR.

#### 3.2.3 Trends from 1990 to 2021

The number of deaths from HT-CKD attributable to low physical activity increased from 1,013 cases in 1990 to 4,479 cases in 2021, representing a 342.15% increase. Similarly, the corresponding age-standardized death rate showed consistent growth, increasing by 54.05%. The number of DALY cases and the ASR followed the same pattern. DALY cases rose from 18,881 in 1990 to 77,879 in 2021 (312.47% increase), while the ASR increased from 0.593 to 0.952, reflecting a 60.54% growth. During the study period, the global age-standardized death rates and DALYs presented significant upward trends with the EAPCs 2.76% (95% CI: 2.20–3.34%) for death rates and 2.65% (95% CI: 2.22–3.08%) for DALYs (Tables 3, 4). An analysis of annual trends in deaths and DALYs from 1990 to 2021 demonstrated a consistent year-on-year growth pattern (Figure 2).

#### 3.2.4 Gender subgroup analyses

During the study period, global trends by gender demonstrated that the number of deaths and DALYs cases of HT-CKD attributable to low physical activity increased annually in both men and women. Specifically, global deaths due to HT-CKD attributable to low physical activity increasing from 492 (95% UI: 77–1,281) in 1990 to 2,015 (95% UI: 421–4,963) in 2021 among men, and from 521 (95% UI: 78–1,382) in 1990 to 2,464 (95% UI: 540–5,974) in 2021 among women. This represented an approximately4.1-fold increase for men and a 4.7-fold increase for women (Figure 3; Table 3). The patterns of DALYs mirrored those of deaths, with an approximately 3.3-fold increase [from 9,364 (95% UI: 1,272–27,078) to 35,873 (95% UI: 6,438–95,864)] for men and a 4.1-fold increase [from 9,517 (95% UI: 1,272–28,246) to 42,006 (95% UI: 7,874–105,694)] for women during the same period (Figure 3; Table 4). The age-standardized death rates and DALYs exhibited consistent upward trends over the study period, with EAPCs of 1.11 (95% UI: 1.03–1.20) in men and 2.03 (95% UI: 1.81–2.24) in women for death rates and of 1.21 (95% UI: 1.14–1.29) in men and 2.1 (95% UI: 1.92–2.27) in women for DALYs (Tables 3, 4). These findings highlight the sustained and significant annual increases in both mortality and disease burden attributable to HT-CKD due to low physical activity across both genders.

#### 3.2.5 Age subgroup analyses

From 1990 to 2021, the number of deaths and DALYs of HT-CKD attributable to low physical activity exhibited a consistent upward trajectory with advancing age. This pattern was consistent across both genders (Figure 4; Tables 3, 4). In 2021, the 85–89 age group recorded the highest numbers of deaths and DALYs for both males and females. Female deaths surpassed male deaths in this age group. Regarding DALYs, peaks occurred in the 80–84 age group for males and the 85–89 age group for females, with females again exhibiting higher rates. An exception was the 70–74 age group, where males experienced slightly higher DALYs when compared to females. ASRs of deaths and DALYs exhibited a consistent upward trend from 1990 to 2021. The ≥95-year age group showed the highest rates, with deaths increasing from 5.471 (95% UI: 1.092–12.66) to 8.567 (95% UI: 2.260–19.680), marking a near 1.6-fold increase, and DALYs climbing from 52.863 (95% UI: 10.862–119.335) to 76.395 (95% UI: 20.170–173.574), indicating a 1.4-fold increase. This group also had the highest growth rates in mortality (EAPC: 3.18; 95% UI: 3.02–3.34) and DALYs (EAPC: 2.84; 95% UI: 2.70–2.97). In contrast, the 80–84 age group exhibited the lowest growth rates for deaths (EAPC: 1.24; 95% UI: 1.08–1.39) and DALYs (EAPC: 1.09; 95% UI: 0.94–1.25). The 40–44 age group showed the most significant increase in deaths (EAPC: 3.56; 95% UI: 3.39–3.74) and DALYs (EAPC: 3.55; 95% UI: 3.41–3.69). These analyses reveal gender-based disparities in low physical activity-attributable CKD burden across different age groups (Figure 4; Tables 3, 4).

#### 3.2.6 SDI subgroup analyses

From 1990 to 2021, all SDI groups exhibited significant increases in both absolute numbers and ASRs of deaths and DALYs of HT-CKD attributable to low physical activity. Middle SDI regions demonstrated the largest absolute growth in deaths, rising from 320 (95% UI: 51–848) to 1,603 (95% UI: 395–3,720), whereas low SDI regions showed the smallest increase [86 (95% UI: 13–216) to 221 (95% UI: 41–535)]. DALYs followed identical patterns, with middle SDI regions increasing most substantially [4,861 (95% UI: 647–14,491) to 23,149 (95% UI: 3,869–63,144)] and low SDI regions least [1852 (95% UI: 231–5,037) to 4,906 (95% UI: 768–13,012)].

Regarding ASR trends, high SDI regions had the steepest increase in mortality rate (0.03 to 0.06/100,000), whereas low-middle SDI regions showed the highest DALYs ASR growth (0.477 to 0.979/100,000). In contrast, middle-high SDI regions exhibited the smallest ASR changes for both deaths (0.041 to 0.046/100,000) and DALYs (0.042 to 0.047/100,000). Growth rate analysis exhibited high SDI regions with the highest EAPCs [deaths: EAPC 1.53 (95% UI: 1.33–1.73); DALYs: 2.67 (2.53–2.82)], while low SDI regions had the lowest EAPCs [deaths: EAPC 0.14 (0.01–0.26); DALYs: 0.14 (0.05–0.23)] (Figure 5; Tables 3, 4).

#### 3.2.7 Forecasting the future burden of HT-CKD attributable to low physical activity

The ARIMA and ES models were employed to project trends in deaths and DALYs from HT-CKD attributable to low physical activity during 2022–2050 (Figure 6). According to ARIMA projections, deaths and DALYs would increase for both genders over this period —rising by 41.27 and 56.73% for men and by 49.08 and 77.10% for women, respectively. ASRs exhibited divergent trajectories: death ASRs were projected to decline (men: −3.08%; women: −21.28%), whereas DALY ASRs would increase (men: 39.06%; women: 38.53%). Gender-specific analysis revealed that men experienced smaller increases in absolute deaths/DALYs as well as a less pronounced decline in death ASRs when compared with women, although DALY ASRs increases were similar across genders. In contrast, the ES projected escalating deaths and DALYs for both sexes from 2020 to 2040, followed by stabilization through 2050. Death ASRs were projected to decline steadily (2022–2040) then stabilizing (2040–2050), while DALY ASRs remained largely constant after 2040. In contrast, both models consistently demonstrated two key patterns: absolute deaths and DALYs were lower in men than women, and all corresponding ASRs were persistently higher in men.

## 4 Discussion

This study represented the first comprehensive global burden assessment of HT-CKD attributable to two pivotal modifiable metabolic risk factors—elevated BMI and low physical activity—from 1990 to 2021, with projections to 2050. This analysis demonstrated that elevated BMI is the predominant modifiable metabolic risk factor for HT-CKD burden, accounting for 179,788 deaths and 4.26 million DALYs globally in 2021. These data reflect staggering increases exceeding 390% in deaths and 320% in DALYs since 1990. While contributing a smaller absolute burden, physical inactivity also exhibited concerning growth (>340% increase in deaths/DALYs), indicating its significant role. Crucially, this burden is characterized by profound inequalities: it disproportionately affects older populations, shows geographic hotspots, and reveals gender complexities. Projections to 2050 forecast continued increases, particularly for elevated BMI.

The trend of elevated BMI is becoming increasingly prevalent, with the number of obese individuals increasing steadily each year. Obesity-induced gut microbiome dysbiosis contributes to hypertension through dual inflammatory and metabolic pathways, ultimately leading to insulin resistance, endothelial dysfunction, and progressive organ damage. Microbial metabolites, such as branched-chain amino acids and reduced short-chain fatty acids, further exacerbate metabolic inflexibility and systemic inflammation, establishing a vicious cycle that sustains hypertension (14). These obesity–microbiome interactions converge to elevate blood pressure and intensify renal vascular stress, thereby promoting glomerular hypertension, tubulointerstitial injury, and progression to HT-CKD.

This analysis revealed pronounced age and gender disparities in the burden of HT-CKD attributable to elevated BMI. The burden increases after age 40 and peaks in individuals aged ≥80 years, likely reflecting the compounding effects of cumulative metabolic comorbidities and age-related decline in renal compensatory capacity (15). Pertaining to gender disparities, the male burden predominates in mid-life (e.g., DALYs peak at 60–64 years), while a marked reversal occurs in older women (≥80 years). The reversal of burden predominance in older women aligns with established sex hormone-mediated pathophysiology. Estrogen confers cardiorenal protection in premenopausal women through integrated mechanisms, including enhanced nitric oxide bioavailability, increased cyclic adenosine monophosphate (AMP), suppression of the RAAS and vasoconstrictors, and reduced vascular stiffness (16), which collectively act to maintain lower BP and attenuate renal injury. Postmenopausal estrogen depletion abolishes these protective effects, accelerating arterial stiffness, salt sensitivity, and glomerular hypertension-amplifying CKD progression (17). A higher testosterone-to-estradiol ratio in postmenopausal women is associated with increased risks of cardiovascular disease, coronary heart disease, and heart failure (18). This explains the disproportionate increase in HT-CKD burden among women ≥80 years, coinciding with prolonged exposure to estrogen-deficient states. Indeed, the observed burden disparity is likely further amplified by sex-specific differences in body composition and fat distribution across the lifespan. Evidence suggests that visceral adipose tissue mass is consistently higher in men across all age groups and increases more steeply with age compared to women, even after adjusting for BMI. This contributes to the elevated mid-life HT-CKD burden in men (19). In addition, men demonstrate lower physical activity engagement and greater salt sensitivity when compared with premenopausal women (20). Men also exhibit a greater propensity for alcohol-related weight gain, particularly from high-calorie beer and social drinking patterns. Furthermore, male obesity is significantly influenced by social networks, where acceptance of weight gain among peers can normalize obesogenic behaviors (21). Therefore, strengthening early screening and effective management of chronic diseases in high-risk populations is crucial. Policymakers should incorporate gender differences into intervention design: for middle-aged and young men, strategies should focus on controlling risk factors such as smoking, drinking, and high-salt diets; for elderly women, priority should be given to renal function monitoring and metabolic syndrome management. Additionally, advancing dietary improvements in the elderly should be prioritized to reduce the disease burden (22).

This study demonstrated that age-standardized mortality rates and age-standardized DALY rates varied substantially across regions in 2021, with distribution patterns closely linked to regional socioeconomic status, population structure, and exposure to risk factors. North Africa and Saudi Arabia exhibit the highest global age-standardized mortality and DALY rates for HT-CKD related to elevated BMI. This is primarily associated with the globally high prevalence of type 2 diabetes mellitus (T2DM) and hypertension among populations with elevated BMI in these areas, coupled with low rates of achieving target blood glucose and blood pressure control, which collectively accelerate the progression of CKD (23). Furthermore, the scarcity of primary healthcare resources leads to low early CKD diagnosis, resulting in the highest global age-standardized mortality and DALY rates for HT-CKD in this region. The high-income Asia-Pacific region and Finland have significantly lower age-standardized DALY rates for HT-CKD when compared with other regions. This is primarily due to the widespread implementation of early screening programs (e.g., community-based kidney injury screening for high-BMI populations), standardized secondary prevention interventions (e.g., intensive blood pressure/glucose management), and a high concentration of medical resources (e.g., accessible specialized nephrology treatment centers) (24). In 1990, low SDI regions bore the heaviest age-standardized disease burden due to weak basic medical facilities and inadequate control of risk factors. However, by 2021, high SDI regions experienced the largest increase in age-standardized mortality rate, likely linked to intensified population aging and increased susceptibility to age-related kidney injury. In contrast, low- and middle-SDI regions experienced the most significant growth in age-standardized DALY rates, primarily caused by rapid urbanization. The widespread adoption of high-salt/high-sugar diets, coupled with reduced physical activity, contributed to an increase in elevated BMI levels, thereby accelerating the progression of diabetes and hypertension (25).

Low physical activity is increasingly becoming the global norm, driven by sedentary jobs, screen-based entertainment, and car-centric urban design. This study found that the number of deaths and DALYs due to HT-CKD attributable to low physical activity continued to increase amid intensifying population aging. Notably, the growth rate has slowed in recent years, potentially reflecting increased public awareness of exercise, widespread health promotion of balanced diets, and strengthened primary healthcare interventions (26). In 2021, deaths and DALYs from HT-CKD due to inactivity increased steadily from age 40 and peaked at ≥80. This pattern is likely attributable to age-related declines in renal reserve function, where reduced physical activity accelerates glomerular hyperfiltration and tubulointerstitial fibrosis. Additionally, physical inactivity negatively impacts muscle mass and function in individuals aged 60 and older, and reduced muscle mass (sarcopenia) further impairs renal metabolic function (8, 27, 28). Hypertension and physical inactivity act synergistically to promote CKD progression, while age-related declines in vascular elasticity and more pronounced abnormalities in renal hemodynamics further exacerbate these effects in the elderly population (29). A randomized clinical trial demonstrated that supervised physical activity interventions significantly improved blood pressure control in hypertensive subjects, reinforcing the value of structured activity programs in high-risk populations (30). Furthermore, physical activity confers prognostic benefits in hypertensive individuals, including those with established left ventricular hypertrophy, without inducing adverse cardiac remodeling (31). Gender-stratified analysis shows that the peak of disease burden occurs slightly earlier in men than in women (peaking in men aged 60–64 years and women aged 65–69 years), though overall mortality and DALY levels in men are slightly lower across all age groups. This discrepancy is closely associated with hormonal differences (e.g., the potential pro-inflammatory role of testosterone in men vs. the vasculoprotective effect of estrogen in women) (32, 33), occupational exposure (men have a higher proportion of physically demanding jobs, partially offsetting the impact of insufficient activity), and age-related physiological changes (the decline in vascular protective mechanisms post-menopause is more pronounced in women) (33). Additionally, regular moderate-to-low-intensity physical exercise can significantly improve renal outcomes in CKD patients and should be prioritized as a primary strategy for HT-CKD prevention and management (34). Supporting this finding, a 2024 cohort study found that increased physical activity was associated with improved kidney function when assessed using cystatin C-based eGFR (35). From a regional and national perspective, the disease burden exhibits significant geographical heterogeneity, with its distribution patterns were closely linked to regional socioeconomic status, population structure, and lifestyle characteristics. Importantly, low physical activity and elevated BMI often coexist and may interact synergistically to exacerbate HT-CKD risk. Evidence suggests that physical inactivity amplifies the adverse metabolic effects of obesity, including insulin resistance and systemic inflammation, thereby accelerating renal injury. Promoting physical activity may partially mitigate obesity-related cardiorenal damage, even in the absence of significant weight loss (36, 37).

Promoting moderate-to-low-intensity physical exercise confers significant health benefits for patients with CKD, particularly among the elderly. Appropriate physical activity enhances NO bioavailability, inhibits the release of inflammatory cytokines, and reduces oxidative stress responses, thereby effectively preventing endothelial dysfunction (38, 39). In addition, exercise improves aerobic capacity and overall functional status, thereby enhancing patients’ subjective quality of life score. Additionally, it synergistically optimizes blood pressure control and improves hematological parameters (e.g., hemoglobin levels). Currently, the most widely recommended regimen is 30 min per day, 3 to 5 times per week (34). The intensity and frequency should be adjusted to the patient’s tolerance, ensuring safety while maximizing benefits.

This study employed a dual-model forecasting strategy, utilizing both ARIMA and ES models, to project the future burden of HT-CKD attributable to elevated BMI and low physical activity. The ARIMA model assumes that the underlying time series can be rendered stationary through differencing, with future values expressed as a linear function of past observations and past error terms. While ARIMA is effective in capturing complex temporal structures like autocorrelation and trend, its performance may be suboptimal in the presence of sudden, non-linear shifts or when the data-generating process changes over the forecast horizon (13). In contrast, the ES model assigns exponentially decreasing weights to historical data, making it highly adaptive to recent changes. However, this very characteristic can also be a limitation, as it may overemphasize short-term fluctuations and underperform in identifying stable long-term seasonal patterns, particularly when the series exhibits significant volatility or lacks a clear trend (13). The GBD study employs a sophisticated and standardized methodology to address data sparsity and missingness, including extensive data synthesis from diverse sources, spatial–temporal Gaussian process regression, and cause of death ensemble modeling to generate estimates for all locations, even those with limited or no direct data. While this process aims to minimize bias and produce the most complete picture of global disease burden, it inevitably introduces uncertainty. This uncertainty is quantified and presented in the GBD study as UIs. The forecasting models inherit this underlying uncertainty, and we did not perform additional imputation for missing data, as the input data from GBD are already modeled estimates that account for data gaps.

In light of the projected rise in global burden of HT-CKD associated with elevated BMI and low physical activity, urgent implementation of targeted public health interventions is required to address these modifiable risk factors, particularly obesity and physical inactivity. Evidence suggests that multimodal strategies, combining lifestyle modification, pharmacotherapy, and policy-level changes, are most effective in achieving sustainable weight loss and improving cardiorenal outcomes (40). For high-risk groups, including older adults and populations in low- and middle-income countries, culturally tailored interventions that promote Mediterranean or Paleolithic dietary patterns, regular physical activity, and cognitive-behavioral support have demonstrated promise in reducing obesity and its metabolic sequelae (41). At the policy level, taxation of sugar-sweetened beverages, restrictions on marketing of unhealthy foods, and improved access to anti-obesity medications and bariatric surgery represent effective measures to curb the obesity epidemic (42). Furthermore, integration of digital health tools and telemedicine may enhance the scalability and adherence of lifestyle interventions in underserved regions. Future public health initiatives should prioritize equitable access to these interventions in order to mitigate the growing burden of HT-CKD, with particular emphasis on vulnerable populations.

This study has several limitations. First, reliance on aggregated GBD data restricted subnational analyses (e.g., state/province-level variations in disease burden), potentially obscuring localized risk patterns and intervention priorities. Second, the diagnostic complexity of HT-CKD—requiring sustained blood pressure monitoring and renal function assessment—may lead to the underestimation of burdens in low-SDI regions where healthcare access is limited. Furthermore, the potential for misclassification exists, as the ICD-based case definition for HT-CKD may include patients with concurrent conditions such as diabetes, thereby complicating the precise attribution of CKD etiology. Third, the 2050 forecast assumes constant risk exposure and healthcare trends, overlooking potential disruptions from emerging therapies, policy shifts, or climate impacts that could alter metabolic risk trajectories. Fourth, residual confounding is inevitable in GBD’s risk attribution framework, where synergistic interactions between elevated BMI, physical inactivity, and unmodelled factors (e.g., diet quality and air pollution) may significantly affect HT-CKD burden accuracy. Fifth, estimates, particularly in regions with limited underlying data, are characterized by wide uncertainty intervals, which should be considered when interpreting the findings. Sixth, the analysis was conducted at the overall HT-CKD level without any differentiation between disease stages, which precludes insights into whether the observed burden is primarily driven by early-stage incidence or progression to advanced stages.

## 5 Conclusion

Our analysis demonstrated a substantial and progressively increasing global burden of HT-CKD associated with elevated BMI and low physical activity. Among these risk factors, elevated BMI exerted a remarkably greater impact. In 2021, elevated BMI contributed to 179,788 deaths and 4.26 million DALYs globally, representing 0.26% of global mortality and 6.17% of DALYs. In contrast, low physical activity contributed to 4,479 deaths and 77,879 DALYs. Both risk factors were significantly associated with increases in absolute deaths, DALYs, and ASRs from 1990 to 2021, as evidenced by substantial percentage increases and significant positive EAPCs globally and across most subgroups. The burden exhibited pronounced geographical heterogeneity, with the highest ASRs consistently observed in North Africa and the Middle East, and the lowest in parts of Asia and high-income regions. The United States consistently exhibited the highest absolute burden. Gender disparities showed higher absolute burdens in men for elevated-BMI-attributable HT-CKD, whereas women showed faster ASR increases for low physical activity. Age analyses exhibited burdens peaking in older populations (≥80 years), with the ≥95 age group experiencing the highest rates and growth. SDI analyses indicated significant increases across all quintiles, with high SDI regions showing the steepest ASR growth rates for elevated BMI and low SDI regions the slowest. Forecast for 2050 using ARIMA and ES models indicated continued increases in absolute deaths and DALYs attributable to both risks, particularly elevated BMI. However, trends in ASRs varied between models and genders. These findings underscore elevated BMI and low physical activity as major, modifiable metabolic drivers of the growing HT-CKD pandemic, necessitating urgent and targeted public health interventions worldwide. To mitigate this escalating burden, policymakers should prioritize national strategies that integrate fiscal policies, foster environments conducive to physical activity, and strengthen primary healthcare systems to ensure early screening and effective management of obesity and hypertension. Interventions must be designed to address regional and demographic disparities. For example, strategies should focus on metabolic syndrome in the ageing population with high-SDI, while enhancing basic healthcare capacity in low-SDI regions. Gender-specific approaches should address lifestyle factors in men and postmenopausal renal protection in women. Implementing these coordinated actions is essential to mitigate the growing HT-CKD pandemic driven by modifiable metabolic risks.

## Data Availability

Publicly available datasets were analyzed in this study. Data can be obtained from the following website: http://ghdx.healthdata.org/gbd-results-tool.
